# Psychosocial assessment of adolescents and young adults in paediatric hospital settings: patient and staff perspectives on implementation of the e-HEEADSSS

**DOI:** 10.1186/s12913-023-09621-2

**Published:** 2023-06-22

**Authors:** Daniel Waller, Sophie Bailey, Elham Zolfaghari, Jane Ho, Daniela Feuerlicht, Kirsty Ross, Katharine Steinbeck

**Affiliations:** 1 Sydney Children’s Hospitals Network, Sydney, Australia; 2 The University of Sydney, Sydney, Australia; 3 New South Wales Department of Health, Sydney, Australia; 4 South Eastern Sydney Local Health District, Sydney, Australia; 5 Faculty of Medicine and Health, University of Sydney, Camperdown, NSW 2006 Australia

**Keywords:** Adolescence, Youth, Young Adult, Health, Psychosocial Assessment, Implementation science, Health services, HEEADSSS

## Abstract

**Background:**

The main causes of morbidity and mortality for adolescents and young adults are preventable and stem from psychosocial and behavioural concerns. Psychosocial assessments can help clinicians to identify and respond holistically to risks and strengths that may impact upon a young person’s physical and mental health. Despite broad support at a policy level, the implementation of routine psychosocial screening for young people remains varied in Australian health settings. The current study focused on the pilot implementation of a digital patient-completed psychosocial assessment (the e-HEEADSSS) at the Sydney Children’s Hospital Network. The aim of this research was to evaluate patient and staff barriers and facilitators to local implementation.

**Methods:**

The research used a qualitative descriptive research design. Semi-structured interviews were conducted online with 8 young patients and 8 staff members who had completed or actioned an e-HEEADSSS assessment within the prior 5 weeks. Qualitative coding of interview transcripts was carried out in NVivo 12. The Consolidated Framework for Implementation Research guided the interview framework and qualitative analyses.

**Results:**

Results demonstrated strong support for the e-HEEADSSS from patients and staff. Key reported facilitators included strong design and functionality, reduced time requirements, greater convenience, improved disclosure, adaptability across settings, greater perceived privacy, improved fidelity, and reduced stigma for young people. The key barriers were related to concerns over available resources, the sustainability and continuity of staff training, perceived availability of clinical pathways for follow-up and referrals, and risks related to off-site completions. Clinicians need to adequately explain the e-HEEADSSS assessment to patients, educate them about it, and make sure that they receive timely feedback on the results. Greater reassurance and education regarding the rigour of confidentiality and data handling procedures is required for patients and staff.

**Conclusions:**

Our findings indicate that continued work is required to support the integration and sustainability of digital psychosocial assessments for young people at the Sydney Children’s Hospital Network. The e-HEEADSSS shows promise as an implementable intervention to achieve this goal. Further research is required to determine the scalability of this intervention across the broader health system.

**Supplementary Information:**

The online version contains supplementary material available at 10.1186/s12913-023-09621-2.

## Introduction

Health risks and behaviours that present during adolescence and young adulthood (12 to 25 years) can have profound and cumulative impacts across the life-course, influencing long-term physical and mental wellbeing, the development of chronic conditions, educational achievement, socio-emotional development, and the attainment of life goals [1, 2]. In 2020, four of the top five leading causes of health burden for young Australians were related to mental health or behavioural concerns [3]. Such issues can contribute billions of dollars of long-term cost to the Australian economy through lost productivity effects, welfare payments, forgone tax, and direct health expenditure [4]. Addressing the psychosocial factors relevant to adolescents and young adults’ wellbeing is therefore critical [5–8].

Psychosocial risks are often clustered: some young people live with multiple risk factors [5–7, 9, 10]. It is important that hospital services working with young people are supported to recognise and respond early to complex presentations with multiple risk factors. Psychosocial assessments can identify strengths and risks for young people and support interventions that promote current and future physical and mental health. However, getting young patients to volunteer information that impacts their health and wellbeing can be a challenge for a number of legitimate reasons [11]. This has led to both a focus on improving clinician skills for communicating with young people [11] and the promotion of psychosocial assessment tools to support holistic approaches to healthcare [5–7, 12–14].

A well-regarded such assessment for adolescents and young adults is the HEEADSSS assessment [5–8, 15, 16]. This assessment was initially conceptualised as a developmentally appropriate tool to facilitate communication and empathy between clinicians and young people, as well as to create a confidential and respectful space where young people can disclose information perceived as being important to their lives. The assessment progresses from addressing less sensitive topics to more emotionally charged issues, as outlined by the acronym: **H**ome, **E**ducation and employment, **E**ating and exercise, **A**ctivities, hobbies, and peer relationships; and progressively moves to more sensitive topics such as: **D**rugs and alcohol, **S**exual activity, sexuality and gender identity, **S**uicide, self-harm, depression, mood, and sleeping patterns, and **S**afety and spirituality [5, 6, 8, 17]. Traditionally, the HEEADSSS assessment has been administered as a face-to-face semi-structured interview, allowing health professionals, even those with limited training in youth engagement, to identify underlying issues that affect the physical and mental health and wellbeing of young people [5–7].

In Australia, state governments and peak medical bodies have recommended use of the HEEADSSS assessment for young people attending community and hospital health services; particularly if it is the first time they present [13, 18–20]. Despite broad support at a policy level [13, 18–21], the implementation of routine psychosocial screening of young people using the traditional HEEADSSS assessment remains varied in Australian health settings [7, 12, 14, 15]. Posited barriers to implementing psychosocial assessment include clinician’s lack of familiarity and confidence with assessments, and staff and patient time pressures [12, 14, 22]. Identifying methods to improve uptake (i.e. health professional and patient utilisation) and implementation of the HEEADSSS assessment would be beneficial [12, 14].

### The e-HEEADSSS

A digital patient-completed version of the HEEADSSS assessment (referred here on as the e-HEEADSSS) has recently been developed with a strong focus on ‘digital empathy’ to put the patient at the centre of the experience [23–25]. Hosted on the commercial Tickit Health platform [25], the e-HEEADSSS features a grade-4 literacy level and allows young people to complete an assessment via a digital device (e.g. smartphone, tablet) with privacy and at their own pace. A summary report of their responses to the assessment are uploaded and available in real-time, in a password protected application available via the internet.

Tickit Health is Service Organization Control 2® compliant and is routinely audited by third parties for risks and adherence to the policies and procedures employed to ensure the protection of the data. Data security is supported by firewalls, intrusion detection, multi-factor authentication, 256 bit Advanced Encryption Standard encryption, and external penetration tests. Tickit Health Australia clients are served out of an Australian based data centre with data encrypted and stored on servers physically located in the same country (or jurisdiction) with backup files stored separately. Tickit Health adheres to Australian personal health information security and privacy standards including the Australian Privacy Act 1988 [26]. Access to Tickit Health is made to staff members via an organisational subscription with individual training and orientation provided.

To date, research on the e-HEEADSSS appears positive. An independent feasibility study (n = 80) published by Tickit Health found that 92% of young people found the e-HEEADSSS easy to understand and use [25]. In 2019, members of our research team [12] published the results of a study pilot that aimed to adapt this tool to the local context at the Trapeze transition service at Sydney Children’s Hospital Network (SCHN). Results demonstrated that the e-HEEADSSS is quickly completed by young people, eliciting higher rates of disclosure for important concerns (e.g. experience of sexual violence) when compared to semi-structured interviews [12]. Patient survey questions from the Tickit Health platform demonstrated high levels of acceptability and ease of use [12]. Similarly, research at a Western Australian regional pediatric hospital found the e-HEEADSSS increased adolescent inpatient uptake rates from 12 to 54% when compared to the traditional face-to-face HEEADSSS assessment interview [14]. Disclosure rates were again higher when using the e- HEEADSSS in this study [14]. These results show that the e-HEEADSSS substantially improves the uptake, fidelity, and effectiveness of psychosocial assessment of young people within health settings [12, 14, 25]. This provides the impetus for a focus on broader implementation within the Australian heath system.

### Implementation setting

The SCHN is the major provider of tertiary paediatric care within New South Wales, Australia [27, 28]. The network incorporates two large university-affiliated paediatric hospitals which offer inpatient, outpatient, and emergency care to their respective local communities as well as metropolitan, regional and rural referral sites [28]. These are two of only three paediatric hospitals in the entire state of New South Wales.

In 2021, work began to integrate the e-HEEADSSS into the SCHN Electronic Medical Records (eMR) system. The assessment is made accessible to patients by a clinician creating an eMR order that sends a link via email or text to a digital device (personal or supplied on-site). Part of this integration process involved the SCHN implementation team deciding on the creation of an appropriate number of ‘red flags’ for specific concerns that that should be prioritised for follow-up and potential referral by clinicians. Table 1 outlines the e-HEEADSSS items that generate a red flag within the Tickit Health system and the eMR for SCHN users.

**Table 1:** Red flag items for SCHN e-HEEADSSS assessment

• I have been forced to do something sexual against my will before • I have tried to or thought about harming myself • Ending my life has been on my mind lately • I [don’t] feel safe at home • I would like to talk more about feeling safe	

In October 2021, a business project was conducted at the SCHN to support uptake of the e-HEEADSSS within selected services including Emergency Departments (ED), the Adolescent Medicine Unit (AMU), and the Trapeze transition service. This work involved the development of policy guidelines and staff educational resources to guide/support use of the e-HEEADSSS. This included clinician online training sessions and web-based information that explained why and how to implement, and additional supports if red flags were raised. Importantly, one of these sites (Trapeze) had already been involved in a previous study of the e-HEEADSSS where a small retrospective analysis was conducted to compare the face-to-face and the digital versions of the HEEADSSS assessment [12]. Assessment of progress within these services is necessary to ensure that the goals of the intervention are achieved, problems are addressed, and resources are used efficiently. In-depth evaluations and implementation studies are warranted to facilitate broader implementation and sustainability of the e-HEEADSSS within the health system. A thorough understanding of youth and staff perspectives is critical to determine the acceptability, feasibility, sustainability, and adaptability of this intervention [22, 29, 30].

### Current study

The primary objective of this research was to qualitatively evaluate staff and patient barriers and facilitators to the implementation of the e-HEEADSSS assessment at selected sites (ED, AMU and Trapeze) within the SCHN. The research question for this study was: What are the key staff and patient-reported facilitators and barriers to implementation of youth psychosocial assessment using the e-HEEADSSS at SCHN? We utilised the well-established Consolidated Framework for Implementation Research (CFIR) [31] to identify barriers and facilitators to implementation across five major domains: (I) Intervention characteristics, (II) Outer setting, (III) Inner setting, (IV) Individual characteristics, and (V) Processes. Organisations can utilise the CFIR to systematically capture thorough, comprehensive, and timely knowledge of barriers and facilitators to practice transformation [32]. This allows adjustments and refinements to be made to an intervention through continuous quality improvement initiatives [32].

## Methods

### Design

The study incorporated a qualitative descriptive research design which complements exploratory research [33, 34] and is reported in accordance with the consolidated criteria for reporting qualitative research (COREQ) [35]. Semi-structured interviews were conducted with hospital patients and hospital clinicians to evaluate current implementation of the e-HEEADSSS. An a priori sample size of 8 patients and 8 staff members was established for this pilot research based on Malterud’s guidance on sample size sufficiency in qualitative studies [36].

Separate interview guides for patients and staff were developed based on the five principles (intervention characteristics, outer setting, inner setting, individuals characteristics, and processes) of the Consolidated Framework for Implementation Research (CFIR) [31]. These guides were iteratively discussed and revised amongst the author team. Key areas of focus for the interviews were staff perceptions of barriers and facilitators to implementation, and patient satisfaction with the assessment and follow-up processes. Addressing these domains within the interview questions allowed for exploration into perceived benefits and disadvantages of the e-HEEADSSS assessment and examination of how it is currently implemented within the local setting. Ethics approval for the study was granted through the SCHN Human Research Ethics Committee (HREC: 2022/ETH00805). All methods were carried out in accordance with relevant Sydney Children’s Hospital Network guidelines and regulations. Informed consent was obtained from all participants and/or their legal guardian(s).

### Eligibility

Table 2 outlines the study eligibility criteria for participation. Staff were eligible to participate if they were a clinician who actioned an e-HEEADSSS assessment within the prior 5 weeks through the ED, AMU, or Trapeze services at the SCHN. Patients were eligible to participate if they were aged 14 to 25 years and had completed the e-HEEADSSS assessment within the prior 5 weeks through the ED, AMU, or Trapeze services at the SCHN. Patients under 18 years who wished to participate were required to have parent/guardian consent to participate. The study excluded patients who were non-English speaking and those with an intellectual disability because the interviews involved complex open-ended questions related to patient’s experiences. Patients deemed at high-risk (e.g. recent experience of physical/sexual assault) were also excluded to avoid any risk of further trauma. Patients younger than 14 years were excluded based on research ethics and governance feedback from the Sydney Children’s Hospital Network.

**Table 2:** Study inclusion and exclusion criteria

Patients	Staff
Patient completed HEEADSSS assessment in past 5 weeks	Staff member actioned an e-HEEADSSS assessment for a young patient in past 5 weeks
**Inclusion**	
Consenting patient at the Sydney Children’s Hospital at Westmead aged 18 to 25 years of age	Staff member from Trapeze, Adolescent Medicine Unit, or Emergency Department at the Sydney Children’s Hospital Network
Assenting patient at the Sydney Children’s Hospital at Westmead 14 to 17 years of age with parent/guardian consent	Staff member over 18 years of age
**Exclusion**	
Patient and/or guardian declines to participate Individuals with intellectual disability Non-English speakers Patient deemed high-risk by study team	Staff member declines to participate

### Recruitment and sample

Recruitment of eligible patients and staff was achieved though convenience sampling [37]. Potential participants were identified through the Tickit Health database and SCHN eMR and were contacted by the research team via telephone (young people) or email (staff). Individuals interested in participation were then emailed the appropriate participant information sheet and an informed consent/assent form.

Parents/guardians of patients under 18 years were offered the opportunity to participate in the research interview alongside the young person under their care but none in the study sample opted to take up this opportunity. Patient participants were also offered a $50 gift voucher to recompense their time contributions for study involvement. Individuals who returned a signed consent/assent form were provided with an interview time and a password-protected Zoom™ meeting link. Links to the meetings were only shared with the interviewer and interviewee. We invited 16 patients (50% response rate) and 30 staff members (26.6% response rate) to participate before reaching our recruitment goal (i.e. 8 patients and 22 staff did not respond within the recruitment timeframe). The 50% response rate for patients was as expected for this age cohort [38, 39] whilst the 26.6% response rate for staff likely reflects the mode of recruitment communication (a single email) and the busy work schedules of this group.

### Interviews

One-on-one interviews were conducted online via a secure and password protected Zoom™ meeting by three researchers (Bailey, Zolfaghari, and Waller). Researchers collectively rehearsed interviews prior to data collection to ensure consistency. The researchers conducting interviews with patients (Zolfaghari and Waller) held qualifications and experience relevant to working with young people that present with psychosocial concerns.

### Data management

Audio recordings from the qualitative interviews were generated by Zoom™ and were saved onto an access-limited and firewall protected folder on the internal drives at SCHN. The de-identified audio files were downloaded and removed from Zoom™ and transferred to be transcribed by an external company, Pacific Solutions ^Pty Ltd^. A confidentiality and data management agreement was in place, meeting HREC and health network requirements. Once received, transcripts were checked against audio files and saved in the access-limited and firewall protected folder on the SCHN drive. Data used for recruitment of the sample were saved in a re-identifiable and password protected format. Qualitative data from interviews were managed, stored, analysed, and presented in a de-identified manner.

### Data analysis

Qualitative analysis of the interview transcripts was carried out by two researchers (Bailey and Waller) iteratively in NVivo 12 [40]. The CFIR [31] was utilised as the main framework to guide deductive coding of participant responses and to identify, examine, and report on themes and patterns [41–43]. Inductive coding [41–43] was used to organise qualitative responses not sufficiently covered by the CFIR factors [31]. All themes generated were discussed and reviewed by the research team. After discussion a final set of themes were agreed upon and defined. Researchers maintained notes throughout the qualitative analysis process.

## Results

Tables 3 and 4 outline the characteristics of the patient and staff participant samples. We recruited 6 patients from the ED (50% response) and 2 patients from Trapeze (33.3% response). The mean age of participating patients (n = 8) was 15.8 years with a standard deviation of 1.4 years. We recruited more female patients to the study (6 female: 2 male) although this gender difference appeared to reflect the pool of eligible patients invited (12 female: 3 male: 1 transgender female).

**Table 3:** Patient characteristics

Participant	Age (years)	Gender	Service	Location	Device	Parental assistance
*Patient 1*	17	Male	Trapeze	Home	Mobile	Some
*Patient 2*	14	Female	ED	On-site	Mobile	None
*Patient 3*	16	Male	ED	On-site	Mobile	None
*Patient 4*	15	Female	ED	On-site	Mobile	None
*Patient 5*	14	Female	ED	On-site	Mobile	Some
*Patient 6*	15	Female	ED	On-site	Mobile	None
*Patient 7*	17	Female	Trapeze	Home	Mobile	Some
*Patient 8*	15	Female	ED	On-site	Mobile	None

**Table 4:** Staff characteristics

Participant	Service
*Staff 1*	AMU
*Staff 2*	Trapeze
*Staff 3*	Trapeze
*Staff 4*	ED
*Staff 5*	ED
*Staff 6*	ED
*Staff 7*	Trapeze
*Staff 8*	ED

We recruited 4 staff members from the ED (17.4% response), 3 from Trapeze (60% response), and 1 from the AMU (60% response). Age, gender and role data for staff are not reported here to protect anonymity and confidentiality of participants.

Qualitative coding of interviews confirmed that the CFIR provided a strong framework to determine implementation barriers and facilitators for the e-HEEADSSS. Table 5 summarises the key CFIR implementation barriers and facilitators identified by participants. Importantly, some discussion points were relevant to multiple CFIR factors and are thus represented as barriers and/or facilitators across multiple domains. Staff discussed implementation issues through themes related to the CFIR factors of individual characteristics, intervention characteristics, inner settings, outer settings, and processes [31]. Patients recounted their experience of the e-HEEADSSS in terms of individual characteristics and intervention characteristics [31]. Qualitative analyses indicated convergence and saturation of themes for both samples, suggesting that our results are valid and generalisable to the current implementation setting. The following results section is organised under these CFIR domains and their sub-factors. In-text illustrative quotes are provided for commonly reported CFIR barriers and facilitators and/or issues that represent risks for health services. A non-exhaustive list of illustrative quotes from participants for all the discussed CFIR domains is available as a supplementary file.

**Table 5:** CFIR barriers and facilitators for e-HEEADSSS implementation

CFIR Domain	Facilitators	Barriers
*I. Intervention characteristics*		
* Design, quality, & packaging*	Email and text invitations (P, S) Minimal time requirement (P) User friendly design (P, S) Aesthetics & layout (P, S) EMR integration (S) Functionality (P, S) Modular design (P)	Confidentiality and data handling concerns (P, S) Technology faults/malfunctions (S) Blunt/confronting questions (P) Length/item repetition (P) EMR crashes (S)
* Adaptability*	Staggered completion of modules (P) Adaptable to different services (S) Off-site completion (P)	Risk with off-site completions (S) Forced choices (P)
* Complexity*	Convenience (P, S) Ease of use (P, S)	Patient risk and liabilities (S) Perceived time burden (S) Follow-up procedures (S) Return of results (S) Patient factors (P, S)
* Relative advantage*	Improved accuracy/sensitivity (P, S) Reduced time requirement (S) Reduced social interaction (P) Increased response rates (S) Increased disclosure (P, S) Improved data capture (S) Improved objectivity (S) Perceived privacy (P, S) Improved fidelity (S) Built-in feedback (S) Convenience (P, S) Reduced stigma (S)	Confidentiality and data handling concerns (P, S) Technology faults/malfunctions (S) No immediate clinician support (S) Risk with off-site completions (S) Reliance on device access (P) No non-verbal cues (P, S) Multi-step processes (S) Impersonal (S)
***II. Outer setting***		
* Patient needs & resources*	Thorough understanding of patient needs (P, S)	Informed consent and explanation (P) Feedback to patient (P)
* Cosmopolitanism*	Essential services connected (S)	Lack of awareness of available external connections (S) Preference for internal support (S)
***III. Inner setting***		
* Networks & communications*	Connections with supervisors and mental health teams (S)	Perceived lack of, or difficulty establishing, clear clinical pathways and protocols (S)
* Available resources*	Private spaces and designated consultation times (S) Education and online resources at roll-out (S)	Sustainability/continuity of training (S) Perceived risk of unavailability of referral/support services (S)
* Relative priority*	Perceived importance of intervention (S) Leadership (S)	Perceived scope of practice (S) Competing demands (S) Time constraints (S)
***IV. Individual characteristics***		
* Knowledge and beliefs*	Positive attitudes and beliefs towards intervention (P, S)	
***V. Process***		
* Engaging*	Effective initial roll out by implementation team (S) In person training at roll-out (S) Online resources (S)	Sustainability/variability/continuity of training (S)
* Executing*	Private spaces and consultation times (S) Potential for built-in reminders (S) Role fit (S)	No private spaces or consultation times (S) Variability of health settings (S) AMU preferences/ uptake (S) Staff turnover (S)

* P = Patient, S = Staff

### Intervention characteristics

Design, Quality, and Packaging refers to the perceived quality in how an intervention is designed and presented [31]. All participants complimented the visual aesthetics, appeal, accessibility, and functionality of the e-HEEADSSS. Patients found it easy to navigate and liked that it was presented in modules that centred on specific themes. They reported no computing lags or delays. The main implementation barrier cited by patients was the number and repetitiveness of assessment items. One patient cited concerns over online data capture and patient confidentiality. Nevertheless, all patients were satisfied with the assessment and would recommend it for other young people.

“The survey itself was good, it was appealing. I liked how it looked.” – Patient 1.

Staff described the platform as user-friendly and discussed how the app-like format is relevant and familiar to adolescents. They found the results page and flagging system conducive for streamlining the identification of issues that may not be the presenting problem, and producing a rapid summary. Key staff barriers related to technological challenges (eMR crashing, assessments sent via text return quicker than email, patients not receiving the link, or eMR integration issues). Staff reported few data confidentiality concerns as once data is on the SCHN eMR system it is subject to network privacy/data protection like any other assessment. However, some questioned the data handling agreement with the third party (Tickit) and highlighted that greater transparency about this may be required during training.

Adaptability refers to the ability of the intervention to adapt, transform, or be reinvented to match the local environment and its needs [31]. Participants praised the ability to complete the assessment online, off-site, and at a time convenient to them. Two patients indicated slight difficulty using forced-choice answers to comprehensively represent their situation. Staff across sites described different ways of using the e-HEEADSSS demonstrating its ability to adapt to local settings. ED clinicians described it as a comprehensive aid to streamline follow-up discussions whereas designated adolescent services’ clinicians (Trapeze, AMU) described it as a brief screening tool that ensured psychosocial histories were accurately recorded. Some staff reported concerns over patient off-site completions and were hesitant to action an e-HEEADSSS for patients in unsupervised, external environments.

“If the young person is at risk of harm and they do it, say at night or when there’s no one around… it may be triggering for them and then it can put them at risk of harm if they don’t know how to access that support at those times.” – Staff 3.

Complexity refers to the perceived difficulty of implementation and usage due to the disruptiveness, intricacy, and number of steps required for the intervention to be effective [31]. Patients and staff identified the ease of use and convenience of the e-HEEADSSS as a facilitator to implementation. Patient-identified barriers included individual factors (e.g. potential comprehension issues, motivation etc.) and subsequent requirements for parent/carer assistance. Staff noted inherent risks and liabilities if red flags are returned and not actioned efficiently. Staff also speculated that uninitiated health workers may view the e-HEEADSSS as complex, uncomfortable, and time-consuming.

#### Relative advantage

Relative advantage captures stakeholder perspectives on the advantage of implementing an intervention versus an alternate or existing solution [31]. Patients noted several advantages of the e-HEEADSSS over the face-to-face HEEADSSS interview. These included greater perceived privacy, increased convenience, improved disclosure, greater accuracy of responses, and reduced requirements for social interaction. Patients indicated that the e-HEEADSSS served as a useful starting point for young people to organise their thoughts before going into an in-person discussion with a health professional that could be potentially confronting, anxiety-producing, awkward, or embarrassing. Potential barriers reported by patients included a reliance on access to an electronic device, the inability of health professionals to monitor non-verbal cues, and reservations over online confidentiality and data handling.

For staff, the most cited advantage of the e-HEEADSSS was the decreased time it takes to administer and address assessment results. Clinicians (particularly those working in the ED) felt the e-HEEADSSS could produce more accurate and comprehensive results than face-to-face assessment within their demanding (i.e., time poor) health settings. Staff also discussed the benefits of the e-HEEADSSS being a standardised set of questions thus removing user variability and improving intervention fidelity. Other advantages highlighted by staff were increased response rates, patient convenience, user friendliness, improved data capture, built-in feedback, perceived patient privacy, and removal of parent or clinician judgement. Potential disadvantages included a lack of immediate (i.e. real-time) clinician support, risks related to off-site completions, impersonal nature of online assessment, lack of non-verbal cues, technological difficulties (eMR capability and compatibility), limited knowledge around data handling agreements, and the multi-step processes involved in actioning the assessment (obtaining a contact, sending the assessment and return processes).

“It was a little bit uncomfortable because it’s a lot of personal information going into it, but I think it was nicer to do it on the website than it would be talking to somebody. I’m not very good at talking to people about my issues, so I thought it was really useful to be able to put it into just my mobile phone instead of telling people all about that.” - Patient 6.

### Outer setting

Patient Needs and Resources describes the extent to which an organisation understands and prioritises patients and their needs [31]. Staff and patients indicated that the SCHN has a strong general understanding of young people’s needs. Staff highlighted several patient factors that influence their decision to action an e-HEEADSSS (e.g. intellectual, psychological, or emotional capacity, tiredness, maturity levels, age etc.). Staff acknowledged the assessment may be triggering or intrusive for young people and emphasised transparency and confidentiality. They highlighted the need for more accessible clinical pathways and protocols to ensure patients’ needs are met if red flags are generated. Some patients were surprised to be given an e-HEEADSSS and reported minimal introduction on what to expect (e.g. data management, informed consent). Some reported limited clinician feedback on their results.

“Are we really doing the right thing by having some concerns raised and not having a clear clinical pathway to deal with them?” – Staff 1.

Cosmopolitanism describes the extent to which an organisation is connected to external entities [31]. Staff-identified facilitators included well-established external connections with relevant stakeholders and services (e.g. parents, general practitioners, psychologists, specialists, youth justice, child protection services etc.). Staff-identified barriers included responsibilities for organising follow-up and knowledge of available external services. Teams internal to the hospital (rather than external agencies) were often the preferred and primary contacts for follow-up/ongoing care requirements informed by the e-HEEADSSS.

### Inner setting

Networks and Communications refers to the interconnectedness and quality of communications within an organisation [31]. Most staff reported being well-connected to other internal health care teams or senior staff. However, some felt these connections and in-hospital communications could be more formal and clearly defined within a clinical pathway specific to that local environment or service. Some clinicians were unsure if e-HEEADSSSS referral protocols currently existed and indicated such agreements could be difficult to set up within a hospital environment.

“If it’s a mental health concern, then we have our mental health team here at all times. If it’s child safety, we have our child protection unit here… and we obviously have supervisors here if we have any questions of which direction we should go in.” – Staff 4.

Available Resources refers to the extent of resources dedicated to implementation and its ongoing success [31]. Staff from outpatient services (Trapeze and the AMU) cited the availability of private spaces and designated consultation time as a facilitator for implementation. Staff also indicated the brief training and online resources provided during the initial roll-out period helped. However, staff did not indicate that there was a standardised education package or commitment to continued education. Whilst staff generally supported the expanded use of the e-HEEADSSS this was identified as a potential risk if resources (and clinical pathways) are not available when patient concerns are identified. Senior staff noted that quality improvement projects or clear data would be required to justify the commitment of further resources for broad implementation of the e-HEEADSSS across the SCHN.

Relative Priority refers to the perceived importance of the implementation within the organisation [31]. Staff highlighted the importance of the intervention as a personal motivator for actioning an e-HEEADSSS and indicated that leadership within their departments also drove implementation. Staff also noted that the SCHN holds a focus on youth-centred care and discussed potential barriers for other services, hospitals, or local health districts. Competing demands, time constraints, and perceived scope of practice were highlighted as potential barriers for broader implementation of the e-HEEADSS.

### Individual characteristics

Knowledge and beliefs about the intervention refers to stakeholder attitudes regarding the intervention, their value placed on the intervention, and their awareness of facts, truths, and principles related to the intervention [31]. Patients indicated e-HEEADSSS offered a helpful snapshot to health professionals to guide personalised treatment. They indicated it helped them organise and think about issues affecting them personally and provided them with agency to raise personal concerns. All patients recommended e-HEEADSSS assessments for young people in health settings regardless of perceived personal need.

Staff attitudes towards the e-HEEADSSS were similarly positive with all participants expressing a desire to complete psychosocial assessments for any young person who presents at their health service setting. The e-HEEADSSS was consistently cited as providing an important opportunity to intervene and improve youth health and wellbeing and to identify areas of strength. Staff discussed the importance of identifying psychosocial concerns and including this in young peoples’ health profiles to holistically inform clinicians.

“It’s good to have that – just that ability to see where young people are at. I’m saying that in a very broad, generalised way, because it is sometimes difficult to gauge that, and also, because, when young people are asked, it’s like, yeah, I don’t really want to talk about it.” – Patient 7.

### Process

Engaging refers to the degree of involvement of key stakeholders in the development, implementation and use of the intervention and strategies to maintain engagement [31]. When discussing engaging, staff emphasised the strength of the e-HEEADSSS launch process across SCHN and the subsequent increase in likelihood to action an e-HEEADSSS assessment, particularly after online training sessions and web education pages. Some concerns over the sustainability, variability and continuity of training and engagement were expressed. Patients did not report engagement for the purposes of implementation. This has been previously noted as a weakness of implementation projects within youth health services [30].

Executing describes the process of fulfilling and completing implementation as planned [31]. Different departments perceived the success of implementation differently. Staff from outpatient adolescent services (AMU, Trapeze) described the e-HEEADSSS intervention a having a natural fit with their role. However, the staff member from AMU noted that the face-to-face nature of their consultations led other clinicians in their service to preference the in-person interview format of the HEEADSSS (rather than the e-HEEADSSS). There was also some uncertainty of current utilisation levels within the ED and senior staff indicated that they felt it still was not a part of regular workflow. This service setting has unique implementation barriers such as lack of private space but most noted was the regular turnover of staff and hence clinician training variability. ED staff described a consistent rotation of paediatric nursing and variability in start dates for junior doctors resulting in a lack of orientation. Staff suggested that more frequent reminders of the available tool would increase usage, for example pop-up reminders on patient notes.

## Discussion

This research aimed to identify key barriers and facilitators to implementing digital psychosocial assessment for young people using the e-HEEADSSS in real-world paediatric hospital settings. This work complements existing research on the efficacy and acceptability of similar approaches and provides both patient and staff perspectives on implementation challenges [12, 14, 44]. A key strength of the study is the utilisation of the CFIR, an implementation science framework that can be used to not only identify implementation issues but also develop tailored solutions [31]. The study thus serves as a useful first step in a needs analysis to inform implementation of the e-HEEADSSS assessment for young people in health settings in Australia.

Overall, our results demonstrated strong support for the e-HEEADSSS from patients and staff. Key benefits of the e-HEEADSSS highlighted by patients and staff include strong design and functionality, reduced time requirements, greater convenience, improved disclosure, adaptability across settings, greater perceived privacy, improved fidelity, and reduced stigma for young people. These perceived benefits appeared to be associated with health professional’s willingness and motivation to implement the e-HEEADSSS in their workplace and patient’s satisfaction and willingness to complete the assessment. Importantly, all the patients in our study recommended the e-HEEADSSS as a useful assessment for other young people to assist with their healthcare journeys. These results provide support to the systematic review findings [22] that indicate that adolescents prefer a self-administered tool over a face-to-face psychosocial interview that requires personal disclosure. Similar to our study, this review found that adolescents feel positively about electronic mental health assessments as they help them in disclosing sensitive information and provide a structure to their thoughts [22]. Together, these findings indicate that the e-HEEADSSS may provide an implementable approach to psychosocial assessment.

The key barriers to implementation of the e-HEEADSSS appear related to the ‘implementation readiness’ of organisations with concerns over available resources, the sustainability and continuity of staff training, perceived availability of clinical pathways for follow-up and referrals, and risks related to off-site completions. From the patient perspective, clinicians need to be mindful to fully inform and educate patients about the assessment and need to ensure that feedback on results occurs (even for those where minimal risks are identified). For both groups, greater reassurance and education regarding the rigour of confidentiality and data handling processes appears to be needed. Importantly, further implementation research can help to better understand these challenges and to develop health service policy and practice solutions.

### Strengths and limitations

It is important to acknowledge the limitations of this research study. First, the study featured a small sample size with only 8 staff and 8 patients interviewed. This could impact the depth and generality of results as it is possible that the limited sample size did not capture the entire range of experience from clinicians or patients’ actioning or completing e-HEEADSSS assessments. Nevertheless, our qualitative analyses indicated that we achieved saturation of themes for our specific implementation setting. We contend that the current study also serves as a strong starting point to better understand implementation issues for the e-HEEADSSS intervention in novel health settings.

Second, our sample did not include staff who have not utilised the e-HEEADSSS or patients who, received, but did not complete an assessment. This sampling bias likely affected the results of our study and may have missed important considerations. Future research should look to include these populations to further understand barriers to utilisation and implementation of the e-HEEADSSS.

Third, our analytical framework employed deductive coding using the CFIR to guide interpretation of results. Whilst it is possible that this approach could have introduced some bias in interpretation, we believe that the utilisation of well-developed implementation science models [31] improved the utility of research findings, particularly when considering how/whether results could translate into future implementation action. Furthermore, we did not restrict coding to our deductive framework with coders left free to create novel inductive codes where appropriate.

Finally, the research focused on a specific group of health services set within a large paediatric hospital network setting. The SCHN incorporates two of the three paediatric hospitals based in New South Wales and it could be argued that the barriers and facilitators to digital psychosocial assessment for young people would differ in other Local Health Districts/Health Services that do not have such a strong focus on youth health. Whilst we acknowledge this as a study limitation, we argue that this only further emphasises the need for research focused on broadening implementation of the e-HEEADSSS across broader settings. Broader implementation of the e-HEEADSSS may require us to address a ‘don’t ask, don’t tell’ philosophy for young people within the health system through education and training on issues surrounding young patient disclosure. On the other hand, promoting e-HEADDSSS as a psychosocial triage may not be so confronting in generalist (i.e. adult health care) where there are stronger links with appropriate community services than occur in specialist, multidisciplinary paediatric care.

### Implications for healthcare

Implementation of the e-HEEADSSS has the potential to shift the current paradigm for adolescent healthcare within Australian hospital settings. By supporting implementation of a new digital method for psychosocial assessment we can improve pathways to mental and physical health management and move towards a focus on holistic care for young people. The improved fidelity, sensitivity, acceptance, and reduced time requirements of the e-HEEADSSS assessment [12] will allow for more young people to be assessed and more psychosocial concerns to be disclosed.

### Directions for future research

Further research studies featuring greater sample sizes are required to identify implementation barriers and facilitators unique to other health service environments. This work along with cost-benefit and effectiveness analyses can inform broader implementation and scaling of digital psychosocial assessments for young people across the Australian health system.

## Conclusions and future directions

The current study lays the groundwork for continued research focused on implementation of the e-HEEEADSS assessment for youth in health settings within Australia. We hope that this work serves to push forward implementation of psychosocial screening for young people. Innovative approaches are required to determine barriers, facilitators, risks, and opportunities from multiple stakeholder perspectives (e.g. patients and families, health executives, health service managers, IT teams, administrators, clinicians etc.) to support integration and sustainability of the e-HEEADSSS into routine practice for young people who come into contact with the Australian health system.

## Electronic supplementary material

Below is the link to the electronic supplementary material.

Supplementary Material 1

## Data Availability

The datasets analysed are not available due to Human Research Ethics requirements. Interview guides are available upon reasonable request from Dr Daniel Waller.

## List of abbreviations

The HEEADSSS: A psychosocial assessment that covers Home, Education and employment, Eating and exercise, Activities, hobbies, and peer relationships and progressively moves to more sensitive topics such as Drugs and alcohol, Sexual activity, sexuality and gender identity, Suicide, self-harm, depression, mood, and sleeping patterns, and Safety and spirituality
The e-HEEADSSS: A digital version of the HEEADSSS assessment
SCHN: Sydney Children’s Hospital Network
ED: Emergency Department
AMU: Adolescent Medicine Unit
eMR: Electronic Medical Records
CFIR: Consolidated Framework for Implementation Research
HREC: Human Research Ethics Committee
