# Supplementary material for: Psychosocial assessment of adolescents and young adults in paediatric hospital settings: patient and staff perspectives on implementation of the e-HEEADSSS
Source: BMC Health Serv Res. 2023 Jun 22;23:683. doi: 10.1186/s12913-023-09621-2 (PMC10288690; doi:10.1186/s12913-023-09621-2)
Supplement: Supplementary file 1 — Supplementary Material 1 [file 12913_2023_9621_MOESM1_ESM.docx]

| **I. Intervention characteristics** | |
| --- | --- |
| Design, Quality, and Packaging | - “The survey itself was good, it was appealing. I liked how it looked.” - Patient 1 - “I think the general format of everything was done well. It was very organised and each category stayed true to a theme which was nice and simple. A few of the questions seemed a bit similar to me so that seemed like a few of them could have been condensed into one.” - Patient 2 - “It was easy to navigate around.” - Patient 3 - “From a software perspective, I thought it was very good. The layout of it was quite good and helpful. There was one part of it, where it had, describe how you're feeling, with a thumbs up sign, and down.” - Patient 7 - I guess I had the confidentiality thing, I didn't really know where it was going, I don't think I was told where the information was really going. I did assume it was just going to the doctor, the health [unclear], that sort of thing. I was not really told where the information was going. - Patient 6 |
|  | - “The ease of the tool is very important, particularly for a group who don't always have the most patience. I think it's quite useful because young people do like to use digital platforms.” - Staff 1 - “It will tell you at the top, which I think is a real benefit, it tells you exactly how many reds [flags] there are, so then that way if you are short time, you can kind of go through all the reds first” - Staff 2 - “They’re very used to doing the whole like app-thing.. it kind of has that app-like setup to it. So it’s almost a little bit therapeutic, like genuinely I kind of see they really enjoy doing it.” - Staff 4 - “Occasionally our computer system crashes. We mostly have notice that that's going to happen. Sometimes, it just happens, which means we resort to paper forms, and obviously we wouldn't be able to use it in those circumstances. I can't think off the top of my head of any other reasons that have crossed my mind that I might not use it.” - Staff 7 - “The whole HEADSS assessment goes on the EMR system. It's the same network issues that would happen with the whole EMR, like the medical health system. I don't know much about the third-party that's bringing in, like the Tickit people, what they do with their data.” - Staff 8 |
| Adaptability | - “It was convenient, I could do it at home.” - Patient 1 - “I did it on my iPhone and it was pretty simple… it was pretty smooth.” - Patient 2 - “Some of the questions were hard to answer. I didn’t know which one. I might fit into two categories, but I could only choose one.” - Patient 4 - “I think maybe if there was the option to have little midpoints, or something, rather than just one, two or three, because… it gives you a clearer view” - Patient 7 |
|  | - “I used it [e-HEEADSSS] very much as a brief screening tool because I always ask a HEEADSSS assessment anyway. I guess my use of it might be a bit different to someone else who was not going to do a HEEADSSS assessment and use it just for that purpose.” - Staff 1 - “All those details about their purpose in life, their parents, their family, who they live with. It’s just a very good handy thing to have to get all that information.” - Staff 2 - I would look at the results before I went back to see them and so they would know that I was planning to come back after I’ve read the result. I do find you have that summary page and then everything in detail and you have that kind of flag section. It does help, so again, in an ED, in a scenario where there can be many things that are distracting you like anything like that, that is flagging, just puts a little bit extra in your mind to pay attention to an answer I think is very useful.” - Staff 4 - “If the young person is at risk of harm, and they do it, say at night or when there’s no one around… it may be triggering for them.” - Staff 3 |

| Complexity | - “It wasn’t very complex. It was simple. So, it felt like anyone of any age could do it.” - Patient 2 - “A few of the questions, I did ask her [Mother], what should I put for this? I'm a bit confused.” - Patient 5 - “I will admit, some of the questions were a little bit confusing.” - Patient 3 |
| --- | --- |
|  | - “If it was done in triage, maybe they would get fed up and leave and then there would be flagged issues. So, I guess that could be an issue because then someone would need to follow up.” - Staff 4 - “Most of the health workforce are not comfortable with the psychosocial emotional side of things. Fair enough they don’t deal with it every day or perhaps they’re not trained adequately. So, I think it’s just most people won’t feel like it’s part of their work.” - Staff 5 |
| Relative Advantage | - “I'm not very good at talking to people about my issues, so I thought it was really useful to be able to put it into just my mobile phone instead of telling people all about that.” - Patient 6 - “It’d be a bit inconvenient if you’re not able to use your phone at the time, for example, but it was pretty easy for me and the connection and everything worked well.” - Patient 2 - “Some of the risks, I guess it's hard to put your very, very personal stuff in a website.” - Patient 6 - “I mean, there's the thing of, what will they do with the data, but it's probably like, okay, so, we'll just look at it.” - Patient 8 - “That's the thing with the online format, that you can tell them what you want to hear. I mean, my practice is always just answer it honestly, because at the end of the day, that's just going to be more helpful.” - Patient 7 |
|  | - “It’s been really helpful, to be honest. The time it takes to actually do a HEADSS assessment without, like a normal HEADSS assessment yourself, especially in an emergency department, is substantial. There has been maybe a couple of occasions it hasn’t worked and the difference in time that I’ve actually had to spend is very significant. We’re talking like maybe 30 to 40 minutes extra and obviously in emergency, when you have so many other competing tasks, it is a massive advantage.” - Staff 4 - “What I find is really useful about the computer one is that… you don’t miss out a section, the kids work through it.” - Staff 4 - “A lot of teenagers are embarrassed to answer certain parts of the HEEADSSS assessment and really quite like the ability to answer the questions in a semi-private way, even though they know we’re going to know what the answers are, they don’t have to say it out loud, so if their parent or carer is in the room, they don’t have to say those things in front of them, or in front of us if they find that confronting.” - Staff 6 - “The advantages are a standardised form, a standard set of questions and which takes some user variability out of it. I think a lot of us, when we're busy, particularly, are not good at remembering acronyms and not good at asking things.” - Staff 7 - “All those details about their purpose in life, their parents, their family, who they live with. It’s just a very good handy thing to have to get all that information without having to spend a lot of time.” - Staff 4 - “It might take away the opportunity to actually form a relationship or spend more time with the young person. But generally, in ED, we are limited with time and limited in the levels of skill.” - Staff 7 - “If they’re at home, I will speak to them first and see kind of just generally how they're going and if they’re fine, I always make sure that there’s someone there, a parent or some kind of guardian with them because I do feel like that is something to be aware of.” - Staff 2 - “It [off-site/home completion] can put them at risk of harm if they don’t know how to access that support at those times, or they don’t feel comfortable for whatever reason.” - Staff 3 |

| **II. Outer setting** | |
| --- | --- |
| Patient Needs and Resources | - “It wasn’t something I was expecting to do whilst we were there, but it was easy.” - Patient 2 - “Oh, well, I knew it was going to ask me questions about home life and everything like that, so I kind of knew. But yeah, it was a bit different to what I expected.” - Patient 5 - “I remember the doctor asking me. I don't remember the name of the doctor, but - and it was pretty straightforward. I was asked and I was given clear instructions on how to do it.” - Patient 3 - “I didn't really know where it was going, I don't think I was told where the information was really going.” - Patient 6 |
|  | - “While we obviously pick the adolescent population, we obviously know there's a big spectrum in maturity levels depending on different things.” - Staff 8 - “Are we really doing the right thing by having some concerns raised and not having a clear clinical pathway to deal with them?” - Staff 1 - “Patient related factors, do they have capacity, that intellectual capacity to do the survey? Do they have the sort of psychological or emotional capacity to do it? - Staff 5 |
| Cosmopolitanism | - “She does feel quite supported by her friends and family, she knows that she can call her psychologist and if they’re unavailable we already had a discussion about possibly ringing other services that might help her at that time, so things like Kids Helpline, Beyondblue and Headspace.” - Staff 2 - If they’re already involved with psychological medicine. If they’re pretty depressed or pretty anxious, they’re already getting support for that.” - Staff 5 |
| **III. Inner setting** | |
| Networks and Communications | - “If it’s a mental health concern, then we have our mental health team here at all times. If it’s child safety, we have our child protection unit here… and we obviously have supervisors here if we have any questions of which direction we should go in.” - Staff 4 - There’s a whole load of different things that would potentially need to be escalated, and I don’t know if there is a specific protocol for any of them, because it’s probably several different protocols - Staff 6 |
| Available Resources | - “I might ensure that there was somewhere that they could be privately talked with.” - Staff 7 - “If I have time, which mostly I do, I’ll go through the whole assessment and see what their strengths are and things like that.” - Staff 2 - “Yeah, so it was an online training session. There might have been a PDF that was included. I can't remember that, sorry. There might have been a PDF that we could refer to - remind us how to use it. I think there were lots of different options to attend different training sessions. It was really easy to work that around our other clinical commitments. I think I missed one session and was able to attend another.” - Staff 1 - “So I didn’t formally get training here to do - like I got taught how to use the HEADSS assessment, but basically I have a mental health background, so I originally came from adolescent medical, surgical and mental health nursing, so I kind of know from there all the parts of the HEADSS assessment and how to talk to young people about the results and things like that.” - Staff 2 - “Because I'm not sure that all staff would necessarily understand or have the same resources that we do. Those would be the things, so standardised implementation, assessment of staff that they've understood education, standardised education, and it would probably need to be an on-going, if you like, recording of data on how many staff had done the training. Staff might need a refresher course, calibration about whether staff were acting on the document in appropriate ways and whether it affected outcomes. I think all of that would be a good quality improvement project.” - Staff 8 |
| Relative Priority | - “Our managers and other team members are always around to support us if we need.” Staff - 3 - “We actually need to be asking these questions otherwise how do we know these very important things about their health and safety? But it’s not always just about risk actually. A lot of these responses throw up young people’s strength. So then again if we come from that strengths-based approach, it’s very positive” - Staff 5 |

| **IV. Individual characteristics** | |
| --- | --- |
| Knowledge and beliefs about the intervention | - “It's good to have that - just that ability to see where young people are at. I'm saying that in a very broad, generalised way, because it is sometimes difficult to gauge that, and also, because, when young people are asked, it's like, yeah, I don’t really want to talk about it.” - Patient 7 - “It helped sort out what you are thinking. It wasn't really necessarily the response that I was needing, it was more the ability to work out all these things for myself… Depending on the person, it could really help them out.” - Patient 3 - “I recommend it especially when you’re at distressed times of your life as it might make it easier. It might make it easier for you to identify what might be upsetting you” - Patient 4 |
|  | - It’s excellent. I think it covers all aspects in a psychosocial and it gives a young person an opportunity to raise issues if they may not have been able to talk to anyone.” - Staff 3 - I think as long as you set the table appropriately to have the conversation, it’s an opportunity for open discussion. I’ve never found a negative outcome from talking about things.” - Staff 6 |
| **V. Process** | |
| Engaging | - “We did use it a lot more regularly when it was launched because it was a new tool that lots of people were using.” - Staff 1 - “Yes, there was absolutely training.” - Staff 3 - “It was more a kind of informal introduction, I guess. It wasn’t like a formal sit-down training. But I think it could be something - it would be something useful to highlight to people maybe at the beginning of when they start working in the emergency department.” - Staff 4 - “It's on the orientation talk schedule. They have a day where they’re training everyone there to roll it out.” - Staff 8 |
| Executing | - “We’ve just got this regular turnover of staff. They tend to stay for a few years and then they sort of have to get new people and then they have to sort of get trained again.” - Staff 5 - “We would use it slightly differently, because we're always going to do a face-to-face HEEADSSS after we meet them in addition to it.” - Staff 1 - “No, we have - so the lead implementer, the staff specialist who is responsible for implementing HEADSS and giving an education package and resources around it, has gone off on family leave and will not be back until January. So we had initial education about how to use it, which was brief, so probably a half-hour session to staff specialists.” - Staff 7 - “Nurses were informed of the process, but haven't yet been, if you like, credentialed to use it, which is the obvious next step. Then we have just embraced it and gone forward.” - Staff 7 |
